# Graves’ Clinical Lecture

**Published:** 1846-01

**Authors:** R. J. Graves


*Clinical Lecture, delivered at the Meath Hospital. Pneumonia in Children.* By R. J. Graves, M.D., &c.—The next case to which I shall call your attention is that of J. Duffy, a little boy, who, on the first of this month, was attacked with symptoms of fever followed by thirst, nausea, and vomiting. On his admission, six days afterwards, we found him complaining of headache and sleeplessness, but the fever, thirst, and abdominal symptoms had disappeared. The weight of the disease seemed to have fallen chiefly on the respiratory system, for he had loud, hard, incessant cough, increased by motion, a suspicion of the existence of pneumonia struck me, and on applying the stethoscope under the right scapula, I found extensive dulness, absence of the respiratory murmur, and crepitus. Over all the rest of the thorax respiration appeared to be clear and natural, but here, instead of the clear sound, we had nearly complete absence of respiration, with slight crepitus. The disease was therefore fever complicated with extensive inflammation of the postero-inferior part of the lung.

Now inflammation of the parenchyma of the lung, when it attacks children, presents some remarkable points of difference from the same disease in the adult.

I am not at present prepared to enter upon an explanation of the pathological differences which exist between the pneumonia of infants and children below the age of three, and children above that age; certain it is that important differences do exist, and that also inflammation of the lungs in adults is essentially different from both. Again, in persons advanced in years a fourth variety of pneumonia occurrs, whose features are very characteristic and peculiar. Observe gentlemen, when I assert that inflammation of the lungs assumes different characters, according to the age of the individual attacked, I am touching on a practical point of the greatest importance, for I have no hesitation in adding that the same mode of treatment is not applicable to any two or three varieties of pulmonary inflammation. Thus mercurial salivation rapidly produced is our sheet-anchor in the pneumonia of adults and persons in the vigor of life, but such a method of treatment cannot be applied either in infants, children, or in old persons. Again, in the pneumonia of children above three years old, nauseating doses of tartar emetic, persevered in with judgment, and combined with bloodletting, leeching, blistering, &c., are chiefly to be relied upon, whereas in infants a perseverance in the exhibition of tartar emetic for more than one or two doses is inadmissible, while minute doses of calomel, ipecacuanha, and chalk, exert a most beneficial influence on the complaint. In infants, too, there must be much more caution exercised with respect to the detraction of blood; but, gentlemen, I find that this subject have not yet been examined with that attention it deserves, and that consequently we cannot lay down general rules for your guidance. Certain I am that much remains still to be done concerning the best means of treating inflammation of the lungs according to the age of the patient. A very good foundation for the pathology of this disease in infants and children has been laid down in an essay by Gerhard, which you will find detailed in a former number of the Dublin Medical Journal.

In the case before us there were some circumstances worthy of remark, as being calculated to make a false impression, and to prevent us from following the only mode of treatment likely to produce relief. There was no febrile action present, and the pulse was only 72, soft and regular. There was scarcely any heat of skin, the bowels were natural, and he had some appetite; the only circumstances indicative of general disturbance of the system were some foulness of tongue and want of sleep. Here the state of the pulse and skin, with the absence of fever, would be likely to mislead the practitioner, and cause him to overlook the real nature of the disease. I need not tell you, gentlemen, that this would be a very important error; the spontaneous efforts of nature would be totally inadequate to its removal, and it would in all probability terminate fatally.

You may perhaps ask, whether the state of pulse, observed in this boy, be a good or bad sign. If we look to nosological arrangements, we shall find that pneumonia is generally accompanied by a strong quick pulse, in fact that this is its natural condition under such circumstances, and therefore we should say that the slow and tranquil state of the pulse in this case, was not a good sign. But my impression is that, *cæteris paribus*, the quieter the pulse in pneumonia, the better. It indicates a disease of less violence, and much more amenable to treatment. The worst cases I have seen, all those cases of young and healthy individuals which terminated fatally in spite of active treatment, were cases characterized by high excitement of the circulation, and where bleeding, leeching, and tartar emetic failed in overcoming the hardness, or abating the velocity of the pulse. Thus in a case which I saw with Mr. M. Colles, and where the patient was a powerful strong man, bleeding after bleeding failed to diminish either the frequency or the hardness of the pulse, neither did the most energetic measures in the slightest degree seem to check or even retard the progress of the inflammation, which, producing a rapid hepatization of one portion of the lung after another, soon proved fatal; so great was the arterial action in this case, that all the superficial veins of the hand had a distinct pulsation communicated to them. On the contrary, when the pulse is soft and slow, the disease, though likely to be latent, and thus give rise to error, is generally under the influence of ordinary treatment. Wherever the pulse remains quick and hard after the employment of free antiphlogistic treatment, your prognosis shoul always be doubtful if not unfavorable.

In treating this boy's case I did not order general bleeding, for no matter where the inflammation may be situated, if it has no effect on the general circulation, if it be unaccompanied by increase of pulse and heat of skin, you may dispense with venesection, for it will not do any good. Here your means of treatment should be cupping, leeches, blisters, and the internal use of antiphlogistic remedies. I have ordered this boy to be leeched and blistered; and to take small doses of mercury. I beg leave to observe here, that in exhibiting mercury for pneumonia in children, or boys under the age of puberty, I do not, as in the case of adults, prescribe it in large doses, or with the view of producing a decided action on the system. In the pneumonia of adults, calomel is given in very large doses (I sometimes give it in scruple doses twice a day), with the view of inducing sudden salivation, because we know from experience, that there is nothing which checks so rapidly the progress of inflammatory action. It is true that mercury will occasionally stop the further progress of pleuritis or pneumonia without having affected the mouth, just as we now and then observe the removal of syphilis without actual salivation. But, generally speaking, when you give mercury to cure syphilitic iritis, common pneumonia, or pleuritis, its action is more favorable and more decidedly curative, when it operates fully on the system, as denoted by the affection of the breath, gums, and salivary glands. This, however, is not the case with respect to children or boys under the age of puberty. Mercurialization has not the same beneficial influence on the pneumonia of early life, as at the adult period, and I believe the same remark will be found to hold good with respect to the aged. Certain it is, that in very young children and infants it is scarcely possible to affect the gums, mouth, and salivary glands, in fact to establish a true sore mouth and fetid breath by means of mercury; with respect to the aged I cannot affirm positively. that the same observation applies, but this I know, that large doses of mercury, calomel for instance, do not cure inflammations in old people with anything like the certainty they display in the inflammations of young adults, or of the middle aged. In the present case I have given calomel in small doses, combined with ipecacuanha. It is my intention to carry it so far as to affect the system, and I have combined it with ipecacuanha because its administration in this way has been found exceedingly useful in the bronchitis and pneumonia of children. We attempted here the resolution of the pneumonia by leeching, blistering, an antiphlogistic regimen, and the use of calomel and ipecacuanha, half a grain of the former and a quarter of a grain of the latter, every fourth hour. This acted twice on the bowels, and it is very probable that this soluble state will continue. Yesterday I ordered eight leeches to the chest, and when the bleeding ceased, eight more were to be applied. To-day, I have ordered twelve more. When you apply leeches to the chest in children, you should not rest content with merely ordering them, you should know how they are applied. There is a vast difference between merely prescribing remedies and seeing them properly employed. It too often happens that this powerful means of checking inflammation is rendered inefficient or even injurious by want of attention on the part of the physician. The leeches are ordered and sent from the apothecary's, but they are applied by an inexperienced mother or a bungling nurse. They are put on one after another anywhere they may chance to take, very seldom over the proper place, and during the whole time they remain on, the child's chest is left quite naked. This needless exposure of the chest is further increased by the habit of applying fomentations after the leeches have dropped off; the child gets fresh cold, and when the physician comes next day, he finds matters worse than before. The best wry of applying them is to put them into a small box made of fresh deal shavings, such as is sometimes used for pillboxes, and place this exactly over the inflamed part. Having thus applied them over a circumscribed space, you may draw the bed clothes gently over the child's chest, and he may remain covered until they are about to drop off, instead of fomentations, order a warm dry sheet or large piece of flannel to be placed over the chest, and in this way you will be able to get a large quantity of blood without exposing the child to the risk of cold. You may perhaps think these observations unimportant and even superfluous, but I am convinced that I have seen lives lost for want of proper care and expertness in the performance of an operation apparently so easy.—*Med. News.*

